# Pain and quality of life in leprosy patients in an endemic area of Northeast Brazil: a cross-sectional study

**DOI:** 10.1186/s40249-016-0113-1

**Published:** 2016-03-07

**Authors:** Victor S. Santos, Jamilly C. V. Santana, Fabrícia D. N. Castro, Laudice S. Oliveira, Julianne C. V. Santana, Vera L. C. Feitosa, Ricardo Q. Gurgel, Luis E. Cuevas

**Affiliations:** Federal University of Sergipe, Rua Cláudio Batista, s/n, Cidade Nova, Aracaju, 49100-000 Sergipe Brazil; Liverpool School of Tropical Medicine, Liverpool, UK

**Keywords:** Leprosy, Pain, Neuropathic pain, Quality of life, Sergipe, Brazil

## Abstract

**Background:**

Pain emerges as a challenge in the treatment of leprosy patients. In this study, we describe the prevalence and type of pain in patients with leprosy, and its effect on patients’ quality of life in an endemic area of Northeast Brazil.

**Findings:**

A cross-sectional survey of 260 patients attending leprosy reference centres in Sergipe, Northeast Brazil was conducted. Individuals were assessed for the presence and type of pain, skin sensory loss, peripheral nerve enlargement, touch and pinprick sensations, mechanical allodynia and nerve palpation. Participants completed the Douleur Neuropathique 4 questionnaire, and we also used the Brief Pain Inventory scale and the World Health Organization Quality of Life-BREF instrument to arrive at our results. One hundred and ninety-five (75 %) patients reported pain, mostly of the neuropathic type. Pain was moderate in 84 (43.1 %) and severe in 94 (48.2 %) participants. The presence of pain was associated with disability (*p* = 0.001), leprosy reactions (*p* = 0.004) and lower quality of life. Most patients with neuropathic pain were treated with steroids, despite their low efficacy for this type of pain.

**Conclusion:**

Pain is highly prevalent among leprosy patients and is associated with low quality of life. Leprosy management should include a systematic assessment of the type of pain a patient experiences in order to provide adequate treatment.

**Electronic supplementary material:**

The online version of this article (doi:10.1186/s40249-016-0113-1) contains supplementary material, which is available to authorized users.

## Multilingual abstracts

Please see Additional file 1 for translations of the abstract in to the six official working languages ot the United Nations.

## Background

More than 250,000 new leprosy cases are reported annually worldwide [1]. Leprosy is a infections disease that causes significant skin and peripheral nerve impairment, physical disability and deformity [2, 3]. Although there is a widespread perception that sensory losses preclude patients to perceive pain [3, 4], this is a frequent complaint of patients attending leprosy treatment centres in Brazil.

The presence of pain affects physical and emotional wellbeing; leads to social isolation, relationship and psychological problems, and an inability to work [5]; and increases health service consultations for patients. Systematic evaluation of adverse effects and type of pain and its effect on quality of life (QoL) is needed to increase awareness and encourage the development of appropriate leprosy management interventions. The concept of QoL denotes the impact that an illness or injury has on a person’s wellbeing. It includes physical and psychological health, social relationships and a person’s interaction with the environment [6].

In this report, we describe the prevalence and type of pain in leprosy patients, and the effect that pain has on patients’ QoL in an endemic area of Brazil.

## Methods

A cross-sectional survey of patients aged >15 years who attended the University Hospital Leprosy Clinic and the Leprosy and Tuberculosis Reference Centre in Aracaju was conducted. These are the two leprosy reference centres in Sergipe, Northeast Brazil. All consecutive patients who visited the centres between February and June 2015, who were receiving multidrug therapy for leprosy or who had been previously treated for leprosy and were returning to the centres for follow-up, were invited to participate. Patients with diabetes, those who drank excessive amounts of alcohol, those who were known to be HIV positive, or those who had mental and physical conditions that could interfere with the assessment of pain were excluded.

Participants were interviewed using a structured questionnaire to obtain demographic and clinical information, disability grade, QoL, and the presence and intensity of pain in the three months leading up to enrolment in the study. Leprosy was classified as paucibacillary or multibacillary (MB), and as tuberculoid, borderline, lepromatous or as having an indeterminate clinical presentation, using the World Health Organization (WHO) classification [7]. Nerves were considered to be affected in the presence of pain or nerve thickening on palpation, loss of sensitivity with the monofilament test or motor impairment [8]. Leprosy reactions were defined as episodes of acute inflammation of skin lesions or nerves (type 1), or the appearance of inflamed cutaneous nodules with/without neuritis (type 2) [7]. Disability grades were assessed using the WHO International Classification of Functioning, Disability and Health: grade 0 indicates that there is no loss of sensation or deformity, grade 1 denotes loss of sensation without deformity, and grade 2 refers to loss of sensitivity and deformities [2, 9].

Individuals were appraised for skin sensory losses and enlargement of peripheral nerves by assessing touch, pinprick sensations, mechanical allodynia and palpation of nerves. Patients completed the Douleur Neuropathique 4 questionnaire in Portuguese [10]. Scores ≥4 were classified as neuropathic pain. We also used the Brief Pain Inventory scale to define pain intensity and the level of the worst pain a patient had experienced the week before enrolment in the study. Scores ranged from 0 to 10, with higher scores indicating greater pain severity and its interference with general activity, walking, mood, sleep, work, interpersonal relations and life enjoyment [11]. Quality of life was evaluated using the WHOQoL-BREF questionnaire, which involves four domains (physical health, psychological health, social relationships and environment) [6].

Sample size was calculated to detect a difference of 20 % in the QoL between cases with and without pain, with α = 5 % and 80 % power. We hypothesised that two-thirds of patients would experience pain, and that a patient with pain would be more likely to have low QoL (60 % and 40 % of patients with and without pain, respectively). A sample size of 260 patients (195 who had pain) was required to examine this hypothesis.

The WHO analysis syntax for SPSS Statistics software package version 20.0 (IBM Corporation, Armonk, NY) was used to calculate QoL scores. The chi-square test was used to compare discrete variables of patients with and without pain. Variables associated with pain in the bivariate analysis were entered into logistic regression models using backwards-stepwise procedures. The Mann–Whitney *U* test was used to assess differences between QoL domains, and Spearman’s correlation coefficients were used to describe the relationship between pain intensity and QoL. *P*-values <5 % were considered to be statistically significant.

The study was approved by the Human Research Ethics Committee at the Federal University of Sergipe (CAAE: 31078114.3.0000.5546). Informed written consent was obtained from all participants. Parents or guardians provided written informed consent for minors.

## Results

Two hundred and sixty patients were enrolled in the study, with 195 (75.0 %) experiencing pain, as shown in Table 1. One hundred and sixty-six (85.1 %) patients with pain had neuropathic pain and 29 (14.9 %) had nociceptive pain. Patients with pain were more likely to be MB (*p* = 0.03), live in rural areas (41/195 vs. 5/65, *p* = 0.01), and experience disability (adjusted odds ratio [aOR] = 2.8; 95 % confidence interval [CI] = 1.5–5.3; *p* = 0.001) and leprosy reactions (aOR = 2.5; 95 % CI = 1.3–4.8; *p* = 0.004). Leprosy reactions were present in 68 % (113/166) of patients with neuropathic pain and 58.6 % (17/29) of patients with nociceptive pain. The pain intensity was moderate in 84 (43.1 %) and severe in 94 (48.2 %) patients. Participants with neuropathic pain had higher pain intensity than those with nociceptive pain [median (interquartile range, IQR), 8 (5–10) vs. 5 (4–7), *p* < 0.001]. Pain affected participants’ general activities (140, 71.8 %), mood (105, 53.8 %), walking ability (98, 50.2 %), work (146, 74.9 %) and sleep (105, 53.8 %). A significant correlation was observed between pain severity and the perception of limitations (*p* < 0.001). Patients with neuropathic pain had been prescribed steroids (126, 75.9 %), analgesics (30, 18.1 %) or anti-inflammatories (10, 6.0 %), but only 28 % of all patients experiencing pain reported that the treatment improved it.

**Table 1:** Characteristics and WHOQoL-BREF domain scores of study participants

Variables	Pain		*p*-value
	Yes (*n* = 195)	No (*n* = 65)	
Age, median (IQR)	49 (36–59)	44 (37–61)	0.94**
Sex, male, n (%)	120 (61.5)	41 (63.1)	0.82*
Rural area, n (%)	41 (21.0)	5 (7.7)	0.01*
MB leprosy, n (%)	159 (81.5)	45 (69.2)	0.03*
Clinical form			
Indeterminate	21 (12.2)	7 (11.7)	0.56*
Tuberculoid	31 (18.0)	16 (26.7)	
Borderline	52 (30.2)	16 (26.7)	
Lepromatous	68 (39.5)	21 (35.0)	
>2 affected nerves, n (%)	37 (18.9)	10 (15.4)	0.51*
Disability, n (%)	131 (67.2)	25 (38.5)	<0.001*
Leprosy reaction, n (%)	130 (66.7)	27 (41.5)	<0.001*
Type 1 reaction, n (%)	75 (38.5)	15 (23.1)	0.04*
Type 2 reaction, n (%)	55 (28.2)	12 (18.5)	0.16*
WHOQoL-BREF domains			
Physical health, median (IQR)	50.0 (35.7–64.3)	71.4 (64.3–76.8)	<0.001**
Psychological health, median (IQR)	66.7 (58.3–75.0)	75.0 (66.7–79.2)	<0.001**
Social relationships, median (IQR)	75.0 (58.3–75.0)	75.0 (66.7–75.0)	0.11**
Environment, median (IQR)	56.3 (46.9–65.6)	62.5 (54.7–68.8)	<0.001**
*Neuropathic pain*	*Yes (n = 166)*	*No (n = 65)*	
WHOQoL-BREF domains			
Physical health, median (IQR)	46.4 (32.1–58.0)	71.4 (64.3–76.8)	<0.001**
Psychological health, median (IQR)	57.3 (66.7–75.0)	75.0 (66.7–79.2)	<0.001**
Social relationships, median (IQR)	66.4 (58.3–75.0)	75.0 (66.7–75.0)	0.04**
Environment, median (IQR)	46.9 (46.9–63.3)	62.5 (54.7–68.8)	<0.001**

*Chi-square test; **Mann–Whitney *U* test

The QoL scores in patients experiencing pain were lower in the physical health (*p* < 0.001), psychological health (*p* < 0.001) and environment (*p* < 0.001) domains than patients without pain, and patients with neuropathic pain had the lowest scores for all domains (see Table 1). Patients with neuropathic pain had lower QoL scores than patients with nociceptive pain in the physical health and psychological health domains (see Table 2).

**Table 2:** WHOQoL-BREF domain scores by type of pain

WHOQoL-BREF domains	Neuropathic pain (*n = 166*)	Nociceptive pain (*n = 29*)	*p*-value*
Physical health, median (IQR)	46.4 (32.1–58.0)	60.7 (48.2-71.4)	<0.001
Psychological health, median (IQR)	57.3 (66.7–75.0)	70.8 (62.5-79.1)	0.04
Social relationships, median (IQR)	66.4 (58.3–75.0)	71.4 (66.7-75.0)	0.17
Environment, median (IQR)	46.9 (46.9–63.3)	59.4 (53.1-68.7)	0.18

*Mann–Whitney *U* test

Pain intensity was associated with decreasing QoL in the physical health (r = −0.34; *p* < 0.001), psychological health (r = −0.23; *p* = 0.003), social relationships (r = −0.15; *p* = 0.03) and environment (r = −0.32; *p* < 0.001) domains.

## Discussion

Pain is highly prevalent among patients with leprosy and the presence of pain is associated with low QoL. There is a well-established association between the severity of the disease (in terms of the number of lesions), deformities and stigma, and low QoL [12, 13]. It has been reported that patients with functional limitations scored lower in the QoL assessment in the physical health and environment domains [7]. Pain decreases psychological [5, 14] and mental wellbeing [2, 9], affects mood and often leads to depression, restricts activities, affects a person’s work and disrupts sleep [15].

In this study, patients with neuropathic pain had lower QoL than patients without pain. This is in contrast to another study that reported that neuropathic pain mostly affects patients in the physical health domain [5]. Perhaps the differences between our study and the other report therefore can be explained by the epidemiological context such as the characteristics of patients being enrolled in the studies and differences in the cultural and socioeconomic context, including the availability of long term rehabilitation and support programmes.

Neuropathic pain had a higher prevalence in this study than expected; most studies report that it’s prevalent in 11 % to 70 % of patients [3–5, 9, 14–16]. This could be explained by the selection of patients in referral centres. In Brazil, uncomplicated cases are diagnosed and treated in health centres and only patients with complications, reactions or disabilities are transferred to referral centres.

In the present study, the presence of leprosy reactions was associated with pain, as previously reported by others [3, 9]. Although it is debatable what it is exactly that determines the predominant type of pain in a patient, nociceptive pain seems to be elicited by hyperexcitation of intact nociceptive fibres, and neuropathic pain is likely due to the persistence of immune-mediated reactions in the somatosensory system of peripheral nerves [3]. The use of steroids is considered to be effective for nociceptive pain, but not for neuropathic pain [3, 9, 14]. However, most patients with neuropathic pain in our study had been treated with steroids, highlighting the urgent need to identify the type of pain for appropriate selection of treatment. Pain management is a key component of patient clinical management and has a direct effect on a patient’s QoL [2].

Our study had several limitations. Firstly, we did not investigate QoL changes over time. The analysis was limited to referred patients, who would have had a higher frequency of complications than those treated at health centres, thus overstating the prevalence of pain among leprosy patients. Our findings therefore reflect the magnitude of the problem of patients attending reference centres like the ones in Brazil.

## Conclusion

Pain is a frequent co-morbidity associated with low QoL in leprosy patients. All leprosy patients should be systematically evaluated to identify the presence and type of pain so that appropriate clinical management can be applied. Longitudinal studies are needed to describe changes in the occurrence and type of pain over the course of treatment and rehabilitation, and its impact on QoL. Because neuropathic pain is very common in leprosy patients and because it has a greater impact on a patient’s QoL than nociceptive pain, this highlights the need for research to identify efficacious treatments appropriate for specific types of pain.

## Additional file

Additional file 1. Multilingual abstract in the six officials working languages of the United Nations. (PDF 278 kb)

## Abbreviations

aOR: adjusted odds ratio
IQR: interquartile range
MB: multibacillary
QoL: quality of life
WHO: World Health Organization
WHOQoL-BREF: World Health Organization Quality of Life-BREF
