# Supplementary material for: Pain and quality of life in leprosy patients in an endemic area of Northeast Brazil: a cross-sectional study
Source: Infect Dis Poverty. 2016 Mar 7;5:18. doi: 10.1186/s40249-016-0113-1 (PMC4780133; doi:10.1186/s40249-016-0113-1)
Supplement: Additional file 1. — Multilingual abstract in the six officials working languages of the United Nations. (PDF 278 kb) [file 40249_2016_113_MOESM1_ESM.pdf]

Translation of the abstract into the six official working languages of the United Nations

الألم ونوعية الحياة لدى مرضى الجذام في منطقة موطونة من شماليّ شرقيّ "البرازيل": دراسة مستعرضة

فيكتور إس سانتوس، "جاميلي سي في سانتانا، فابريسيا دي إن كاسترو، لاوديسي إ أوليفيرا، جوليان سي في سانتانا، "فيرا إل سي فايتوسا، ريكاردو كيو جورجيل، لويس إي كويفاس

الملخص

الخلفية: يبرز الألم باعتباره تحديًا في علاج مرضى الجذام. في هذه الدراسة، نقوم بوصف مدى انتشار ونوع الألم الذي يعاني منه مرضى الجذام وآثاره على نوعية حياتهم في منطقة موطونة في شماليّ شرقيّ "البرازيل".

النتائج: أُجري مسحًا مستعرضًا على 260 مريضًا يرتادون المراكز المرجعية للجذام في ولاية "سيرجيبي" بشماليّ شرقيّ "البرازيل". قُيم الأفراد من حيث وجود الألم ونوعه، وفقدان الحس في الجلد، والتهاب الأعصاب الطرفية، والاحساس باللمس وبالوخز، والألم الخيفي ميكانيكي السبب، وجس الأعصاب. ملأ المشاركون استبيان ألم الاعتلال العصبي 4، واستخدمنا كذلك مقياس جرد الألم المختصر والنسخة الأوجز من أداة قياس نوعية الحياة الخاصة بمنظمة الصحة العاللمية (BREF) لكي نتوصل للنتائج التي توصلنا إليها. أبلغ مائة وخمسة وتسعين مريضًا (75 بالمائة) عن شعورهم بالألم، معظمه من النوع الاعتلالي العصبي. كان الألم متوسطًا لدى أربعة وثمانين مشاركًا (43.1 بالمائة) وحادًا لدى أربعة وتسعين مشاركًا (48.2 بالمائة). اقترن وجود الألم بإعاقة (*القيمة الاحتمالية*=0.001) وباستجابات جذامية (*القيمة الاحتمالية*= 0.004) وبتدني نوعية الحياة. عُولج معظم مرضى ألم الاعتلال العصبي بالمنشطات، بصرف النظر عن نجاعتها الضعيفة في علاج هذا النوع من الألم.

الاستنتاجات: يعتبر الألم واسع الانتشار بين مرضى الجذام، ويقترن بتدني نوعية الحياة. يجب أن تشمل معالجة الجذام تقييمًا منهجيًا لنوع الألم الذي يشعر به المريض حتى يتم تقديم العلاج المناسب له.

Translated from English version into Arabic by Heba Kandel, through

TRANSLATORS WITHOUT BORDERS

巴西东北部流行区麻风病患者疼痛和生命质量的横断面调查

Victor S. Santos, Jamilly C. V. Santana, Fabr ícia D. N. Castro, Laudice S. Oliveira, Julianne C. V. Santana, Vera L. C. Feitosa, Ricardo Q. Gurgel, Luis E. Cuevas

摘要

引言: 疼痛是麻风病治疗中的重要难题。本研究描述了巴西东北部流行区麻风病患者疼痛的发生情况和类型，及其对患者生命质量的影响。

结果: 本研究开展了横断面调查，纳入了就诊于巴西东北部 Sergipe 的麻风病参比中心的 260 例病例。测量了研究对象的疼痛情况及类型、皮肤感觉缺失、末梢神经肿大、触觉和针刺反应、机械性触诱发痛及神经触及情况。研究对象完成了 DN4 量表（Douleur Neuropathique 4 questionnaire）；同时，研究者使用简明疼痛量表（Brief Pain Inventory）及世界卫生组织生命质量量表简化版（World Health Organization Quality of Life-BREF instrument）获取所需信息。175 例（75%）报告了疼痛，大部分属于神经性疼痛。84 例（43.1%）属于中度疼痛，94 例（48.2%）属于重度疼痛。疼痛的出现与失能（*p*=0.001）、麻风病反应（*p*=0.004）及低生命质量相关。大部分神经性疼痛患者使用类固醇治疗，尽管该类药物对神经性疼痛疗效有限。

结论: 麻风病患者疼痛发生率高，且于低生命质量有关。麻风病管理需要纳入患者所承受疼痛类型的系统评价以提供适宜的治疗。

Translated from English version into Chinese by Qian Men-bao, edited by Yang Pin, through

Douleur et qualité de vie chez des malades de la lèpre dans une région endémique du Nord-Est du Brésil : une étude transversale

Victor S. Santos, Jamilly C. V. Santana, Fabrícia D. N. Castro, Laudice S. Oliveira, Julianne C. V. Santana, Vera L. C. Feitosa, Ricardo Q. Gurgel, Luis E. Cuevas

Résumé

Contexte : La douleur est devenue un enjeu majeur dans le traitement des malades de la lèpre. Dans le cadre de cette étude, nous avons décrit la prévalence et le type de douleur dont souffrent des malades de la lèpre ainsi que ses effets sur la qualité de vie de ces patients dans une région endémique du Nord-Est du Brésil.

Observations : Nous avons réalisé une étude transversale réunissant 260 patients se rendant à des centres de référence de traitement de la lèpre à Sergipe, Nord-Est du Brésil. La présence et le type de douleur, la perte sensorielle de la peau, l'épaississement des nerfs périphériques, les sensations au toucher et à la piqûre, l'allodynie mécanique et la palpation nerveuse ont été évalués chez ces individus. Les participants ont rempli le Questionnaire de diagnostic des douleurs neuropathiques en 4 questions et nous avons aussi utilisé le Questionnaire concis sur les douleurs ainsi que l'instrument WHOQOL BREF de l'Organisation Mondiale de la Santé afin d'obtenir nos résultats. 195 patients (75 %) ont fait état de douleurs, de type neuropathique dans la majorité des cas. Ces douleurs étaient modérées chez 84 participants (43,1 %) et sévères chez 94 participants (48,2 %). La présence de douleurs a été associée au handicap (*p*=0,001), à des réactions lépreuses (*p*=0,004) et à une diminution de la qualité de vie. La plupart des patients atteints de douleurs neuropathiques ont été traités par des stéroïdes, malgré leur faible efficacité pour ce type de douleurs.

Conclusion : La prévalence de la douleur est élevée parmi les malades de la lèpre et cette douleur est associée à une faible qualité de vie. La prise en charge de la lèpre doit inclure une évaluation systématique du type de douleur dont un patient souffre afin de lui fournir un traitement adéquat.

Translated from English version into French by eric ragu, through

Боль и качество жизни у больных проказой в эндемичных регионах северо-восточной Бразилии: перекрёстное исследование

Виктор С. Сантос, Джемилли К. В. Сантана, Фабриция Д. Н. Кастро, Лаудиче С. Оливьера, Джулиана К. В. Сантана, Вера Л. К. Фейтоза, Рикардо К. Гургель, Луис Е. Куевас

Реферат

История вопроса: Боль представляет собой сложную проблему в лечении больных проказой. В данном исследовании описывается распространенность и тип болей, которыми страдают такие больные, и их воздействие на качество жизни пациентов в эндемичных районах северо-восточной Бразилии.

Результаты: Осуществлено перекрёстное исследование 260 пациентов, проходящих лечение в специализированных центрах в штате Сержипи северо-восточной Бразилии. У каждого больного определялись наличие и тип боли, потеря чувствительности кожи, гипертрофия периферических нервов, ощущения при касании и укалывании, механическая аллодиния и пальпация нервов. Участники заполнили вопросник №4 по невропатической боли (Douleur Neuropathique 4). Для получения результатов использовались шкала из краткого опросника оценки выраженности болевого синдрома и Краткий опросник ВОЗ для оценки качества жизни. Сто девяносто пять (75%) пациентов сообщили о наличии болей, главным образом невропатического типа. 84 человека (43,1%) жаловались на умеренные боли и 94 (48.2%) – на сильные. Наличие болей ассоциировалось с потерей трудоспособности (p=0.001), лепрозными реакциями (p=0.004) и более низким качеством жизни. Большинство пациентов с невропатическими болями получали лечение стероидными препаратами, несмотря на их низкую эффективность для болей этого типа.

Выводы: Боли широко распространены среди больных проказой и ассоциируются с низким качеством жизни. Для организации адекватного лечения больных проказой необходимо систематически оценивать, от каких болей они страдают.

Translated from English version into Russian by Alena Hrybouskaya, through

TRANSLATORS
WITHOUT BORDERS

Dolor y calidad de vida en pacientes con lepra en una zona endémica del noreste de Brasil: un estudio transversal.

Victor S. Santos, Jamilly C. V. Santana, Fabrícia D. N. Castro, Laudice S. Oliveira, Julianne C. V. Santana, Vera L. C. Feitosa, Ricardo Q. Gurgel, Luis E. Cuevas

Resumen

Antecedentes: El dolor surge como un desafío en el tratamiento de pacientes con lepra. En este estudio, describimos la prevalencia y tipo de dolor en pacientes con lepra y su efecto en la calidad de vida de los pacientes en una zona endémica en el noreste de Brasil.

Hallazgos: Se llevó a cabo una encuesta transversal de 260 pacientes que asistían a centros de referencia en lepra en Sergipe, en el noroeste de Brasil. Las personas fueron evaluadas para determinar la presencia y tipo de dolor, la pérdida de sensibilidad en la piel, aumento en el tamaño de nervios periféricos, sensaciones de tacto y de pinchazo, alodinia mecánica y palpación de nervios. Para llegar a nuestros resultados los participantes completaron el cuestionario Douleur Neuropathique 4 y también utilizamos la escala del cuestionario breve del dolor y el instrumento Life-BREF de la Organización Mundial de la Salud. Ciento noventa y cinco (75%) de los pacientes

reportaron dolor, principalmente del tipo neuropático. El dolor era moderado en 84 (43,1%) y severo en 94 (48,2%) de los participantes. La presencia de dolor se encontraba asociada a discapacidad (p=0,001), reacciones a la lepra (p=0,004) y una menor calidad de vida. La mayoría de los pacientes con dolor neuropático fueron tratados con esteroides, a pesar de su baja efectividad para este tipo de dolor.

Conclusión: El dolor tiene prevalencia alta entre los pacientes con lepra y se asocia a una baja calidad de vida. El manejo de la lepra deberá incluir una evaluación sistemática del tipo de dolor que experimenta el paciente para poder ofrecer un tratamiento adecuado.

Translated from English version into Spanish by Maria Alejandra Aguada, through

TRANSLATORS WITHOUT BORDERS
